# Myeloproliferative blood cancers as a human neuroinflammation model for development of Alzheimer’s disease: evidences and perspectives

**DOI:** 10.1186/s12974-020-01877-3

**Published:** 2020-08-23

**Authors:** Hans C. Hasselbalch, Vibe Skov, Lasse Kjær, Torben L. Sørensen, Christina Ellervik, Troels Wienecke

**Affiliations:** 1 Department of Hematology, Zealand University Hospital, Roskilde, Denmark; 2 Faculty of Health and Medical Sciences, University of Copenhagen, Copenhagen, Denmark; 3 Department of Ophthalmology, Zealand University Hospital, Roskilde, Denmark; 4 Department of Research, Production, Innovation, Roskilde, Region Zealand Denmark; 5 Department of Pathology, Harvard Medical School, Boston, USA; 6 Department of Neurology, Zealand University Hospital, Roskilde, Denmark

**Keywords:** Alzheimer’s disease, Myeloproliferative neoplasms, Essential thrombocythemia, Polycythemia vera, Myelofibrosis, Blood cancer mutations, *JAK2V617F*, *TET2*, Chronic inflammation, Dementia, Thrombosis, Capillary stalling, Cerebral hypoperfusion, JAK1/2-inhibitor, Interferon-alpha2, Interferon-beta, Hydroxyurea

## Abstract

Chronic inflammation and involvement of myeloid blood cells are associated with the development of Alzheimer’s disease (AD). Chronic inflammation is a highly important driving force for the development and progression of the chronic myeloproliferative blood cancers (MPNs), which are characterized by repeated thrombotic episodes years before MPN-diagnosis, being elicited by elevated erythrocytes, leukocytes, and platelets. Mutations in blood cells, the *JAK2V617F* and *TET2*-mutations, contribute to the inflammatory and thrombogenic state. Herein, we discuss the MPNs as a human neuroinflammation model for AD development, taking into account the many shared cellular mechanisms for reduction in cerebral blood, including capillary stalling with plugging of blood cells in the cerebral microcirculation. The therapeutic consequences of an association between MPNs and AD are immense, including reduction in elevated cell counts by interferon-alpha2 or hydroxyurea and targeting the chronic inflammatory state by JAK1-2 inhibitors, e.g., ruxolitinib, in the future treatment of AD.

## Introduction

### The Philadelphia-negative myeloproliferative neoplasms (MPNs)

The Philadelphia-negative myeloproliferative neoplasms (MPNs) including essential thrombocythemia (ET), polycythemia vera (PV), and myelofibrosis (MF) are chronic blood cancers [1] with a long pre-diagnostic phase, spanning up to 15–20 years [2]. In recent years, evidence has accumulated that chronic inflammation is a highly important driving force for the development and progression of MPNs [3–7]. Similar to patients with type II diabetes mellitus (DM), most patients with MPNs experience several thrombotic episodes years before MPN-diagnosis [2, 4], being elicited by elevated activated leukocytes and platelets and an activated dysfunctional endothelium as well [8]. Thus, both the metabolic syndrome and type II DM share several features with MPNs, the ultimate outcome being premature atherosclerosis development with organ dysfunction, and organ failure such as cognitive impairment, memory loss and dementia development, age-related macular degeneration, heart failure, peripheral vascular insufficiency, and chronic nephropathy [3–5, 9].

Taking into account the tight association between chronic inflammation and common comorbidities/multimorbidities in the general population [10] and the MPNs as “A Human Inflammation Model” [3–7], we believe that the MPNs are unique model diseases to elucidate common mechanisms for disease development, including the association between, e.g., MPNs and Alzheimer’s disease (AD), thereby opening an avenue for exploring new treatment targets that may benefit patients with a heavy burden of comorbidities such as patients with AD and the MPNs.

Herein, we argue for neuroinflammation and neurodegeneration being integrated in the CNS-related symptom burden in patients with MPNs. Based upon epidemiological, clinical, molecular, immunological, and genomic studies, we discuss the similarities between a common, classic neuroinflammatory disease—AD—and the myeloproliferative blood cancers, the MPNs. It is hypothesized that AD may be prevalent in MPNs but undiagnosed since the debilitating CNS symptoms are overlooked and misinterpreted as being part of the complex burden of CNS-symptoms consequent to multiple infarctions and impaired cerebral microcirculation in patients with MPNs. Since MPNs are seemingly not orphan diseases but actually prevalent underdiagnosed chronic blood cancers [11] with a long pre-MPN-phase of several years (5-10-20 years) [2]—very similar to AD—studies are urgently needed to explore associations between these two chronic inflammatory diseases. The rationales for such studies will be given below with a discussion of common pathways aimed to improve quality of life and prognosis of patients with these diseases, including not least earlier diagnosis to improve clinical outcomes by earlier therapeutic intervention [3, 4, 9, 12–15]. Such efforts are even more urgent, since no current medical therapies are able to revert the advanced stages of AD and MPNs, characterized by severe dementia and severe bone marrow fibrosis, respectively.

## The MPNs as model diseases for the link between chronic inflammation, neuroinflammation, and Alzheimer’s disease

### The concept

Chronic inflammation is the common link between highly prevalent diseases such as atherosclerosis, the metabolic syndrome, type II DM and cancer, in which the JAK-STAT-signaling and the NF-kB pathways are activated and play a major role in disease progression [10, 16–22]. These pathways are constitutively activated in MPNs due to driver mutations, e.g., the *JAK2V617F* mutation, giving rise to sustained elevation of blood cell counts, which in PV always include elevated hematocrit but often leukocytosis and thrombocytosis as well, in ET always elevated platelet counts and in MF in the early hyperproliferative phase leukocytosis and thrombocytosis and in the advanced stages of MF often pancytopenia [1]. Importantly, circulating leukocytes and platelets are in vivo activated with a marked propensity to adhere to each other (circulating microaggregates giving rise to decreased blood flow in the microcirculation in the brain, retina, heart, lungs, and elsewhere) and to adhere to dysfunctional endothelium [8, 23]. Chronic inflammation is also involved in the huge inflammation-mediated MPN-disease burden [3–7] with a massive CNS-related symptom burden, significantly impairing quality of life [24] and also impacting patients’ overall health and productivity [25]. The CNS symptom burden has been attributed to compromised cerebral microcirculation due to elevated cell counts and activated circulating myeloid cells and microaggregates of leukocytes and platelets [1, 8] which intermittently may plug the cerebral microcirculation as described above.

Based upon the above considerations, we herein use the MPNs as “A Human Inflammation Model” [3–7] and as “A Human Model for a Link Between Myeloid Proliferation and Neuroinflammation” to explore and increase our understanding of causative mechanisms of neurodegeneration—not only in MPNs—but in other chronic inflammatory diseases, which are frequently associated with variably elevated blood cell counts, including the metabolic syndrome, type II DM, cardiovascular disease, chronic obstructive pulmonary disease, inflammatory bowel diseases (IBD), and rheumatic diseases such as polymyalgia rheumatica. Table 1 summarizes similarities between common inflammatory diseases, MPNs, and AD.

**Table 1:** Similarities between MPNs, Alzheimer’s disease, type II DM, chronic inflammatory diseases, and smoking

Clinical	MPNs	AD	Type II DM	Smoking	CI	Comments
Risk of CVE	Increased	Increased	Increased	Increased	Increased	Chronic inflammation is involved in disease pathogenesis in all five disease entities
Risk of CKD	Increased	?	Increased	Increased	Increased	Chronic inflammation contributes in all five disease entities
Risk of PA	Increased	?	Increased	Increased	Increased	PA is well described in smokers and in CI in MPNs, CI has recently been hypothesized to elicit and drive clonal evolution
Risk of VT	Increased	?	Increased	Increased	Increased	CI significantly increases risk of thromboembolic diseases
Risk of MS and type II DM	Increased	?	–	Increased	Increased	A recent study has found an association between MS and ET
Risk of AD	?	–	Increased	Increased	?	Epidemiological studies are ongoing to investigate, whether AD is more common among patients with MPNs.
Risk of COPD	Increased	–		Increased	Increased	Smokers and patients with CI and MPNs have an increased risk of developing COPD
Risk of neuroinflammation	?	–	Increased	Increased	Increased	Neuroinflammation is prone to develop in patients with MPNs due to a chronic inflammatory state with elevated cell counts, in vivo cell activation, and recurrent ischemic cerebral multi-infarctions with chronic cerebral hypoperfusion—one of the hallmarks of AD
Risk of cancer	Increased	?	Increased	Increased	Increased	Smokers have an increased risk of cancer, in particular lung and bladder cancer; MPNs are associated with a 40% increased risk of second cancers. CI precedes several cancers
**Biochemical**						
CI markers	Increased	Increased	Increased	Increased	Increased	Chronic inflammation is the common denominator for elevated inflammatory markers in all diseases and smoking as well
In vivo activation of leukocytes, platelets, and endothelium	Increased	Increased	Increased	Increased	Increased	Chronic inflammation is the common denominator for in vivo cell activation in all diseases and smoking as well
Markers of endothelial dysfunction	Increased	Increased	Increased	Increased	Increased	Chronic inflammation is considered to play a major role for endothelial dysfunction in all diseases and smoking as well
Markers of oxidative stress	Increased	Increased	Increased	Increased	Increased	Chronic inflammation with induction of increased oxidative stress is considered of major pathogenetic importance for organ dysfunction and organ failure in all disease entities
**Molecular pathways**						
JAK-STAT/NF-kB, HIF, NF-E2	Increased	Increased ?	Increased	Increased	Increased	The JAK-STAT, NF-kB, and HIF are activated in both smokers and in patients with MPNs, type II DM, and chronic inflammatory disease patients
**Stalling of cerebral capillaries**	Yes	Yes	?	?	?	Elevated cell counts, in vivo cell activation with adherence of neutrophils to monocytes and platelets, and adherence of these cells to dysfunctional endothelium predispose to stalling of cerebral capillaries and cerebral hypoperfusion

*MPNs* myeloproliferative neoplasms, *AD* Alzheimer’s disease, *DM* diabetes mellitus, *CVE* cardiovascular events, *CKD* chronic kidney disease, *PA* peripheral atherosclerosis, *VT* venous thromboembolism, *CI* chronic inflammatory diseases, *MS* metabolic syndrome, *COPD* chronic obstructive pulmonary disease

## Links between Alzheimer’s disease and MPNs. Alzheimer’s disease—a myeloid blood cell disorder?

Major hallmarks of Alzheimer’s disease include vascular dysfunction and cerebral hypoperfusion, degeneration of neurons, and synapses with the development of senile plaques and neurofibrillary tangles in the brain [26, 27]. Senile plaques are characterized by the abnormal accumulation of amyloid β-peptide (Aβ), derived from the metabolism of the larger amyloid precursor protein, APP, abundantly expressed in platelets which process APP through the same pathways as in the brain. Accordingly, platelets have been used as a model to study pathophysiological aspects for development of AD [28].

## A role of platelets in the pathogenesis of Alzheimer’s disease?

### Platelets are activated in patients with MPNs and in patients with Alzheimer’s disease

Being highly important immunoinflammatory cells, platelets secrete an array of the most potent inflammatory signaling molecules, including chemokines (platelet factor 4, PF4, RANTES, and MIP-1α), interleukins (IL-1β, IL-7, and IL-8), prostaglandins, and CD40L [29].

Upon activation by various platelet agonists, platelets also release a vast amount of Aβ peptides and among these in particular Aβ1-40, which mainly contributes to vascular amyloid deposits, whereas the predominant form in neuronal plaques is Aβ1-42 [28].

The prevailing hypothesis on AD pathogenesis—the Aβ hypothesis—is that brain cells are responsible for Aβ peptide overproduction [30, 31]. An alternative hypothesis has recently been developed, implying that beta amyloidosis is developed from Aβ peptides that spread to the brain from the blood. In this context, platelets are indeed candidates to be involved in AD pathogenesis for several reasons. First, as alluded to above, platelets have relatively high concentrations of APP, being released from platelets upon platelet activation [32]. Second, platelets are also the primary source of Aβ peptide in human blood (∼ 90%) [33]. Importantly, this Aβ peptide is similar to that found in the senile plaques of AD patients—and is similarly increased in patients with familial AD harboring APP mutations [34]. Third, the activated platelets in AD have been shown to retain greater amounts of APP [35] and more platelet adhesion and thrombus formation [36]. Fourth, after experimental thrombosis, Aβ peptide has been detected by immunocytochemistry in and around the blood vessels in the brain, and this peptide is released from platelets [37]. Fifth, an association between microinfarcts and AD pathology has been known for several years [38].

The above mentioned platelet proteins also participate in the transendothelial migration of leukocytes when Aβ peptide promotes an inflammatory stimulus. Thus, the uncontrolled activation of platelets in AD patients ultimately results in a chronic inflammatory reaction in brain vessels, implying a sustained endothelial cell stress, which, in turn, may trigger further platelet activation, thereby creating a self-perpetuating vicious circle with further increase in inflammation load and release of Aβ [39, 40]

**Fig. 1:**
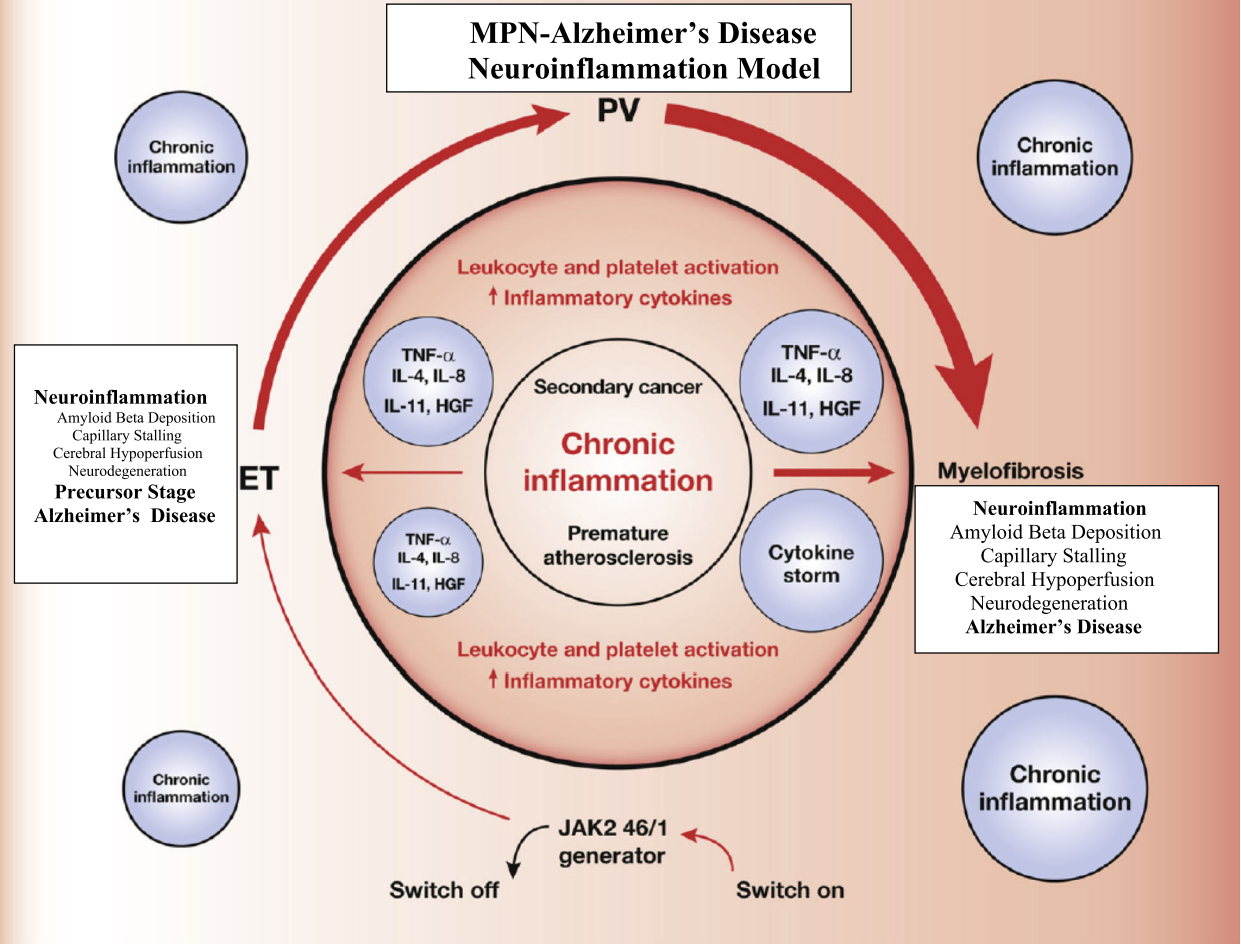
The myeloproliferative blood cancers—essential thrombocytosis (ET), polycythemia vera (PV), and myelofibrosis (MF)—(MPNs) evolve in a biological continuum, spanning 5-10-20 years from the early cancer stages (ET/PV) towards the advanced MF stage. Chronic inflammation is the driving force for this development giving rise to increasing oxidative stress and increasing genomic instability. Inflammatory cytokines drive clonal evolution, and the clone itself generates oxidative stress and inflammatory products which in a self-perpetuating vicious circle elicits more fuel to the fire. In the initial stages of MPNs (ET, PV, and hyperproliferative MF), blood cell counts are elevated (in PV always red blood cells but very often leukocytes and platelets as well, in ET always elevated platelet counts and in some patients elevated leukocyte counts as well and in MF elevated leukocyte and platelet counts). Neuroinflammation is associated with several chronic inflammatory diseases. It is argued that chronic systemic inflammation in MPNs may also elicit neuroinflammation in MPNs and contribute to the CNS-symptom burden in patients with MPNs. It is hypothesized that MPNs are “A Human Neuroinflammation Model” for Alzheimer’s disease development

**Fig. 2:**
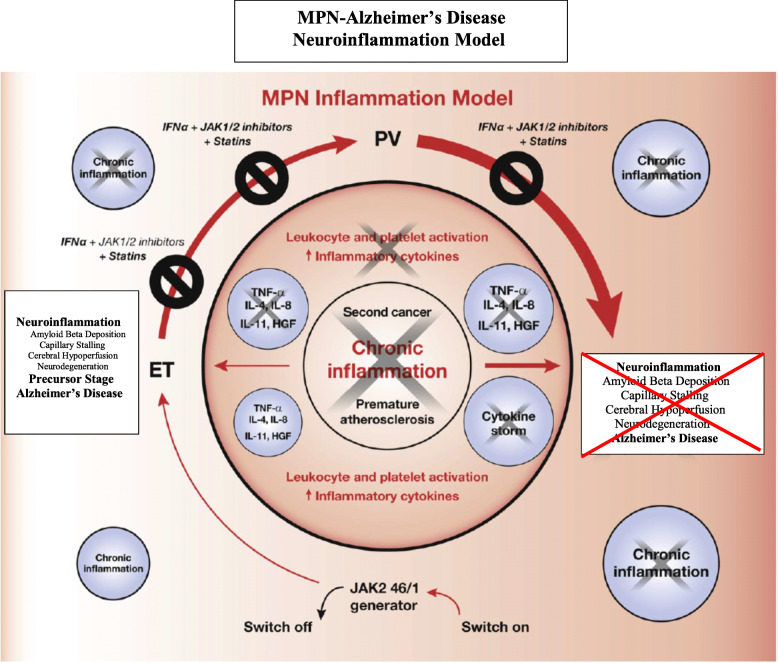
Impact of early intervention with interferon-alpha2 (IFN) + JAK1/2 inhibitor ± statin upon the vicious self-perpetuating circle in MPNs. Early intervention with combination therapy is foreseen to prohibit disease progression by directly targeting the malignant clone (interferon) in concert with dampening of the inflammatory state (JAK1/2 inhibition, statin), which drives the malignant clone and also creates the soil for neuroinflammation and later development of Alzheimer’s disease. From this model, it is envisaged that treatment with IFN and JAK1/2 inhibitor may also be a highly important combination therapy for patients with early stage Alzheimer’s disease

**Fig. 3:**
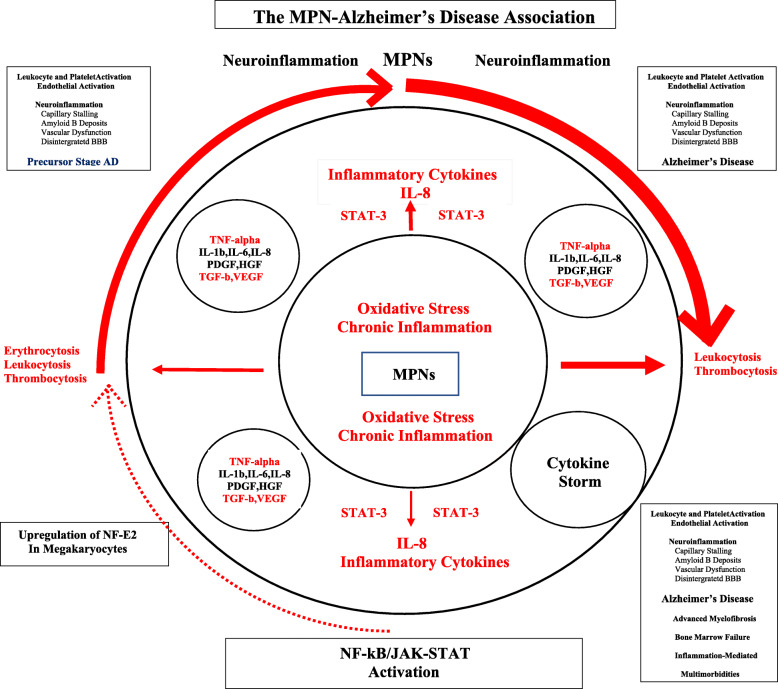
The myeloproliferative blood cancers, MPNs, are associated with chronic inflammation and oxidative stress, which drive the malignant clone from the early cancer stages—ET and PV—towards the advanced burnt-out myelofibrosis stage with bone marrow failure. Smoking is a known risk factor for both MPN- and Alzheimer’s disease (AD) development. By triggering the NF-kB and JAK-STAT pathways, smoking elicits the production of several proinflammatory cytokines, including IL-6, IL-8, and TNF-alpha, which are released from leukocytes and platelets and also give rise to leukocyte-, platelet, and endothelial cell activation. A huge amount of amyloid beta is released from circulating activated platelets. Taking into account that the above pathways are activated in MPNs and AD as well, and the MPNs and AD share a similar inflammatory landscape, it is intriguing to consider, if leukocyte and platelet activation together with chronic inflammation and oxidative stress may constitute the common links, which are determinant for eliciting neuroinflammation, capillary stalling due to plugging of the microcirculation with activated blood cells and ultimately decreased cerebral blood flow and AD development

**Fig. 4:**
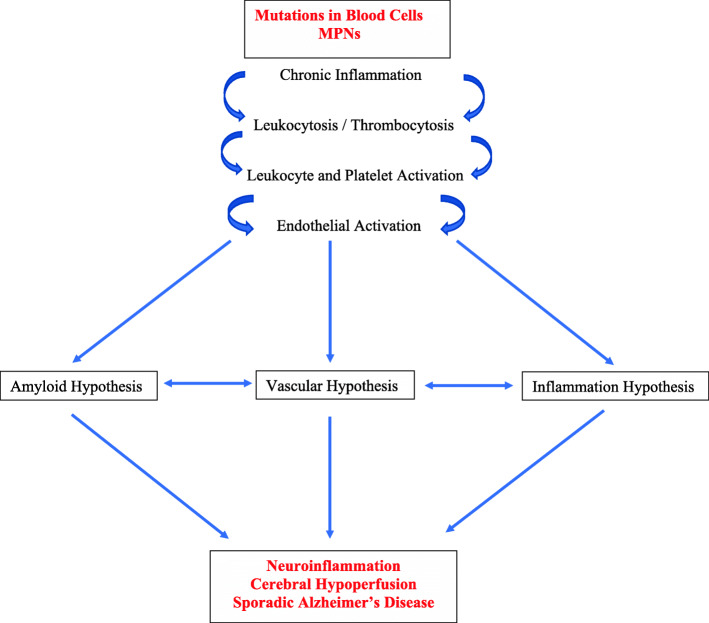
Mutations in blood cells give rise to chronic inflammation and elevated leukocyte and platelet counts, which together with endothelial cells are hyperactivated in MPNs. In polycythemia vera, red blood cell counts are elevated as well. Patients with MPNs and Alzheimer’s disease share several common pathways, which all together unite the different hypotheses of AD, the common denominators being chronic neuroinflammation and cerebral hypoperfusion, the latter being elicited by capillary stalling due to plugging of myeloid cells in the cerebral microcirculation

Elevated platelet counts are one of the hallmarks in most patients with MPNs—at least in the early MPN cancer stages. Due to the driver mutations—*JAK2V617F*, *CALR*, and *MPL*—circulating leukocytes and platelets are also hyperactivated which together with an activated and dysfunctional endothelium elicit a highly thrombogenic condition, which contributes significantly to the huge cardiovascular disease burden. Since platelet activation with release of platelet granule contents, including Aβ and inflammatory cytokines, is considered of paramount importance for AD development [39, 40], it is intriguing to consider that circulating hyperactive platelets in MPN patients—being excessive both in the systemic circulation and in the microcirculation in the brain—might liberate a vast amount of Aβ and inflammatory cytokines in the brain as well. An association between MPNs and development of AD due to an excessive and sustained release of Aβ from circulating activated platelets [39, 40] might deliver additional fuel to the theory that neuroinflammation in AD is elicited and evolves consequent to a chronic systemic inflammatory state [39, 40] which also drives MPN—disease progression in the biological continuum from the early MPN—cancer stages (ET and PV) towards the advanced myelofibrosis stage with bone marrow failure [3–7] and in many patients a huge inflammation-mediated comorbidity burden [3, 4] (Figs. 1, 2, 3, and 4).

## A role of white blood cells in the pathogenesis of Alzheimer’s disease?

### White blood cells—neutrophils and monocytes—are activated in patients with MPNs

Leukocytosis is common in patients with MPNs and associates with an increased risk of thrombosis [1, 8], being explained by several mechanisms, including in vivo activation with aggregation to each other, activated platelets and monocytes, thereby giving rise to circulating microaggregates of activated myeloid cells with ensuing impairment of blood flow in the microcirculation in vital organs, such as the brain, heart, and lungs. A decrease of cerebral blood induces hypoxemia and activation of several signaling pathways which ultimately elicit a neuroinflammatory state with microglia activation and induction of inflammatory cytokines of importance for development of AD (e.g., IL-1beta, IL-6, IL-8, TNF-alpha). Taking into account that persistent leukocytosis with in vivo granulocyte and monocytic activation has preceded the diagnosis of MPNs for several years (5-10-20 years) and leukocytosis in several patients (low risk without prior thrombosis, platelet count < 1500 × 10^9^/L, < 60 years) is not treated, it is intriguing to consider if these chronic blood cancers may predispose to development of neuroinflammatory diseases, such as AD, in which myeloid cells are being increasingly considered of major importance in disease development (Figs. 1, 2, 3, and 4).

Although the significance of leukocytes in the pathogenesis of AD has been addressed in several studies, the literature on circulating cell counts in AD patients is sparse and conflicting; some reporting normal cell counts except for moderate anemia and others elevated neutrophil and monocyte counts [41]. In addition to the above mechanisms, neutrophil extracellular trap (NET) formation may be another highly important thrombogenic factor, both in MPNs, in which the *JAK2V617F* mutation induces NETosis but also in patients with AD [42]. Importantly, in mice, leukocytes have been shown to plug the cerebral microcirculation both in MPN mouse models [43] and in models of AD [44], which will be addressed in detail below. Furthermore, it might be worth speculating if AD with deposition of Aβ in CNS might be explained by defective clearance of excessive Aβ deposition in the CNS due to defective monocytes and defective other immune cells as well [45].

## Do elevated red blood cells (RBCs) in patients with polycythemia vera contribute to development of Alzheimer’s disease?

Elevated RBC counts are associated with an increased risk of arterial thrombosis both in the background population and in patients with MPNs [1]. Red blood cells from patients with PV have been shown to adhere more strongly to endothelial cells. This propensity is mediated by the *JAK2V617F* mutation and may be an additional thrombogenic factor in PV patients but also in the context of PV as a disease, which may predispose to the development of AD.

An impact of RBCs in the pathophysiology of AD is supported by several observations. First, an elevated hematocrit is associated with decreased cerebral blood flow and accordingly cerebral hypoxemia [46]. Second, Aβ binds with high affinity to RBCs and induces oxidative damage to RBCs, which may contribute to the pathogenesis of AD [47]. Third, oxygen delivery to tissues requires that RBCs must be deformable to pass through the microcirculation. When exposed to amyloid beta fibrils, RBCs exhibit decreased deformality. Accordingly, Aβ interactions with RBCs in AD patients may result in impaired oxygen transport and delivery [48].

## Shared cellular mechanisms for reduction in cerebral blood in patients with MPNs and in patients with Alzheimer’s disease?

The MPNs have been named “vascular diseases” due to the sustained prothrombogenic state in the inflamed and activated vascular bed, which also involves the cerebral microcirculation. In PV, the increased number of RBCs contributes significantly to high blood viscosity in the cerebral microcirculation with increased vascular resistance and slowing of blood flow [23, 46]. Similarly, both leukocytosis and thrombocytosis per se may increase resistance in blood vessels and accordingly impair blood flow in several organs, including decreased cerebral blood flow. Transient ischemic infarcts and cerebral infarcts are frequent complications in MPNs due to occlusion of cerebral arteries. Furthermore, obstructions in small vessels might impact neuronal function and elicit acute neurodegeneration even in the absence of symptoms. Importantly, cerebral microinfarcts have been shown to be associated with a more abrupt decline in cognitive function and a higher risk of the development of dementia [49]. In this context, it is important to note that cognitive dysfunction has been demonstrated in patients with ET and PV in the absence of large vessel stroke. In such patients, a dysfunctional microcirculation might account for the neurological symptoms.

Recently, vasculature structure and cerebral blood flow have been investigated in MPN mouse models (*JAK2V617F* transgenic mice with PV, ET, or a mixed MPN phenotype) [43] aimed to describe the effects of excessive blood cells on cerebral microcirculation in ET and PV. A dramatic increase in the fraction of capillaries with stalled blood flow was recorded in MPN mice as compared with control mice [43].

As addressed above, RBCs from PV patients have been shown to have increased adhesion to endothelial cells. High hematocrit values also give rise to abnormally high shear stress on the vessel wall, which may not only facilitate endothelial dysfunction but also activation of leukocytes and platelets in patients with PV. Of note, both leukocyte and platelet activation are typically recorded in patients with PV, altogether facilitating increased adhesion of leukocytes and platelets in the microvasculature [8].

The findings in the MPN mouse models of abnormal stalling of capillaries due to high hematocrit, leukocyte plugs, and platelet aggregates support the clinical observations of cerebral microinfarction in a high proportion of MPN patients—also years before MPN-diagnosis [1, 2].

Indeed, having a large fraction of capillaries stalled may cause chronic brain hypoperfusion, contributing to increase the prevalence of cerebral thrombotic complications in patients with MPNs [8] but most likely also in patients with AD. Thus, cerebral hypoperfusion and microinfarcts are closely linked to cognitive decline in AD development, and silent brain infarcts have been found to double the risk of developing dementia [49]. Patients with silent brain infarcts suffer a broad spectrum of neurological symptoms, including memory deficits, cognitive and behavioral changes with attentional and executive impairments as well as depression [49, 50]—a CNS-symptom burden which by clinical experience is also prevalent in elderly patients with MPNs.

With reference to a reduced cerebral blood flow in patients with MPNs and in MPN mouse models, it is highly intriguing that a most recent novel mouse model study has substantiated that reduced cerebral blood plays a central role in the AD development [44]. This study demonstrated that neutrophils adhere to the walls of capillaries in the cerebral cortex, thereby blocking local blood flow. Importantly, it was also shown that this blockage is the first manifestation of the disease and actually precedes amyloid deposits. Cerebral blood flow was immediately restored after the administration of antibody blocking neutrophil adherence to endothelium in concert with a rapid improvement in the short-term memory tasks in the two murine models. This study further puts in perspective the importance of neutrophils in the development of AD [51] and thereby also the urgent need of exploring MPNs as “A Human Neuroinflammation Model” and the potential role of mutated blood cells giving rise to erythrocytosis with raised blood viscosity, leukocytosis with activated neutrophils and monocytes, and thrombocytosis with activated thrombocytes in AD development (Fig. 4).

## Inflammatory cytokines are elevated and upregulated in patients with MPNs and Alzheimer’s disease

Several studies have investigated the dysregulation of cytokine levels in patients with MPNs [3–7], demonstrating that circulating inflammatory cytokines are elevated with an increasingly dysregulated cytokine profile in the biological continuum from ET and PV to MF as evidenced by whole blood gene expression profiling studies (Figs. 1 and 3) [3–7].

Accordingly, as previously noted, chronic inflammation is today widely recognized as a driver of clonal evolution and significantly contributes to the MPN-symptom burden, including the massive cardiovascular disease burden but also the CNS-related symptoms which may be attributed to “MPN-induced neuroinflammation” (Figs. 1, 2, and 3).

As alluded to above, several lines of evidence support the notion that neuroinflammation may be part of the MPN-associated CNS-symptom complex, including fatigue, anxiety, cognitive dysfunction, and memory loss. Indeed, these symptoms may be the first signals of incipient development of AD in the patient with MPN, but no studies have systematically addressed this highly important issue. Interestingly, patients with AD exhibit elevated peripheral inflammatory cytokine levels very similar to those recorded in patients with MPNs, including elevated C-reactive protein, interleukin-1β (IL-1β), IL-2, IL-6,IL-8, IL-12, IL-18, soluble tumor necrosis factor receptor1 (sTNFR1), soluble tumor necrosis factor receptor2 (sTNFR2), monocyte chemotactic protein-1 (MCP-1), MCP-3, interferon-γ-inducible protein 10, and soluble CD40 ligand. Several of these inflammatory markers have also been found to be elevated in cerebrospinal fluid of patients with AD, including in addition elevated levels of IL-10, transforming growth factor-beta1 (TGFB1), YKL-40, and soluble triggering receptor expressed on myeloid cells2 (TREM2) [52, 53].

## Smoking is a risk factor for the development of MPNs and Alzheimer’s disease

Recently, smoking was suggested as a contributing factor for development of MPNs, taking into account that in both smokers and in patients with MPNs the JAK-STAT and NF-kappaB signaling pathways are activated and both share additionally elevated levels of several proinflammatory cytokines, in vivo activation of leukocytes and platelets, endothelial dysfunction, and increased systemic oxidative stress [54]. Later, several studies have confirmed the association between smoking and MPNs [55].

Several studies have shown that smoking is also a risk factor for development of AD [56], being likely explained by all the factors addressed above for smokers and patients with MPNs, including raised levels of proinflammatory cytokines, in vivo activation of leukocytes and platelets, endothelial dysfunction, and increased systemic oxidative stress which all also are operative in the development of AD (Figs. 1 and 3, Table 1).

## Disintegration of the blood-brain barrier (BBB) and brain vessel angiopathy in Alzheimer’s disease—a consequence of in vivo platelet activation with release of excessive amounts of Aβ and angiogenic growth factors in brain vessels?

As previously addressed, platelet activation may be of particular importance in the context of an association between MPNs and AD. Thus, in patients with MPNs, platelets are constitutively activated, thereby releasing vast amounts of granule constituents, including Aβ and angiogenic growth factors, giving rise to excessive overload of Aβ in the vascular system. In the brain, the excessive release and deposition of Aβ from hyperactive platelets at the endothelium together with release of angiogenic growth factors (e.g., VEGF) likely may disrupt and compromise the blood-brain barrier (BBB) and significantly contribute to the development of cerebral amyloid angiopathy (CAA) as well, thereby adding to the severity of AD, since CAA induces degeneration or destruction of the vessel wall and impairs cerebral blood flow [57].

Since platelets are also activated by Aβ, a vicious self-perpetuating cycle is prone to be established in the cerebral microcirculation with further Aβ release and consequently Aβ-mediated platelet activation and enhanced platelet adhesion on subendothelial matrix proteins. Ultimately, continued recruitment of activated platelets to Aβ deposits may lead to complete blood vessel occlusion. The above scenario has been comprehensively described recently and might be even more exaggerated in patients with MPNs due to high numbers of circulating hyperactivated platelets—a continuous supply of fuel to the vicious circle as described above and accordingly supporting MPNs as “A Human Neuroinflammation for Development of Alzheimer’s Disease.”

## Elevated oxidative stress in patients with MPNs and Alzheimer’s disease

Inflammation generates reactive oxygen species (ROS), and recently the *JAK2V617F* mutation per se has been shown to induce the accumulation of ROS in the hematopoietic stem cell compartment. Using whole blood transcriptional profiling, a massive deregulation of genes involved in oxidative stress and anti-oxidative stress mechanisms has been reported in patients with MPNs with a significant upregulation of several oxidative stress genes in concert with downregulation of important antioxidative defence genes [58]. Oxidative stress is associated with overproduction of several proinflammatory cytokines (e.g., TNF-alpha, IL-1beta, IL-2, IL-6, IL-8, IL-12) which, in turn, can cause oxidative stress in hematopoietic cells in bone marrow and peripheral blood but in other organs as well including the CNS. Recently, oxidative stress has been highlighted as a most important mechanism in the pathogenesis of AD [59].

## Age-related macular degeneration (AMD) is prevalent in MPNs and in Alzheimer’s disease (AD). Is amyloid beta release from activated platelets a contributory mechanism for AMD and AD development in MPNs?

Recently, we have shown that age-related macular degeneration (AMD) is far more common in patients with MPNs than in the background population. Since chronic inflammation is considered of utmost importance for the development of both AMD, AD, and MPNs and complement activation is also involved in AMD and AD pathogenesis, it is intriguing to speculate if chronic inflammation and complement activation are common pathways which associate these diseases. Highly interesting, in vitro stimulation of retinal pigment cells with Aβ1-40, abundant in platelets, and a constituent of drusen has been shown to promote changes in gene expression and cellular pathways [60] which are implicated in the pathogenesis of both AMD, AD, and MPNs, including oxidative stress, inflammation, and angiogenesis. Whether the tight association between AMD and MPNs might also be explained by excessive release of Aβ from circulating hyperactive platelets and if a similar mechanism is operative in the Alzheimer brain as well remains elusive and a topic for future research.

## Down’s syndrome—a model disease for the increased risk of Alzheimer’s disease in individuals with a concurrent risk of myeloid blood cancer

Down’s syndrome (DS) is caused by an extra copy of chromosome 21 which is the most common genetic risk factor for childhood leukemia. Thus, the incidence of acute megakaryoblastic leukemia (AMKL) in children with DS is approximately 500 times higher than that in the general population. A transient myeloproliferative disorder (TMD), which resolves spontaneously, develops in 10% of DS babies, of whom about 20% develop AMKL after several years. Accordingly, during the first years of life, some patients with DS display elevated hematocrit and platelet counts.

Taking into account the abnormal hematopoietic homeostasis in DS patients with a propensity to develop a TMD in childhood and in a subset of these patients after several years AMKL, it is intriguing to speculate if circulating erythrocytes, leukocytes, and platelets—both in early childhood and later—may actually be abnormal, implying an enhanced adherence of leukocytes and platelets to each other and the endothelium in the microcirculation, an enhanced release of intracellular constituents, including Aβ with ultimate deposition in the cerebral microcirculation and cerebral hypoperfusion due to blocking of cerebral capillaries with neutrophils and platelets as previously alluded to. Indeed, a latency period of 10–20 years before AD development in DS patients is compatible with the earlier development of AD in DS—often before the 40th year of life in several patients—and the fact that AD is preceded by a period of 10–20 years, in which patients may be virtually asymptomatic or with only minor cognitive and memory deficits.

Patients with DS exhibit biochemical features of chronic inflammation as evidenced by elevated circulating levels of several inflammatory cytokines and oxidative stress markers. Highly intriguing, a recent study has demonstrated that trisomy 21 causes consistent global changes in the circulating proteome compatible with chronic autoinflammation [61].

Altogether DS is the class type of the consequences of disordered hematopoiesis predisposing to the development of AD. In this context, it is important to note that the abnormal hematopoiesis involves the myeloid cell lineage—and in particular—the megakaryocytic cell lineage with increased production of platelets and release of a huge amount of platelet constituents, which together with blocking of cerebral capillaries with activated leukocytes and platelets may lead to chronic cerebral hypoperfusion—one of the hallmarks of AD. As such, the link between DS, a TMD, AMKL, and AD only adds fuel to the hypothesis and the concept that MPNs—acquired stem cell neoplasms with involvement of the megakaryocyte cell lineage in all MPN-subtypes (ET, PV, and MF)—are “A Human Neuroinflammation Model” for AD development.

## Discussion

Alzheimer’s disease is the most common cause of dementia, being characterized by a decline in cognitive function and neuronal loss [26, 27]. The disease is foreseen to become increasingly prevalent in concert with rising life expectancy. Given the socio-economic burden of AD, the identification of novel mechanisms and predisposing factors for AD pathogenesis and development, as well as novel therapeutic targets [62] is urgently needed.

Herein, we have provided evidence that MPNs are diseases, which constitute “A Human Neuroinflammation Model” and accordingly may be associated with an increased risk of developing AD similar to other chronic inflammatory diseases, such as type II DM. Several lines of evidence support the contention of an association between MPNs and AD. First, the MPNs are associated with mutations in blood cells that give rise to elevated cell counts—red blood cells, white blood cells, and platelets [1], which are associated with impaired cerebral blood flow [23, 46], stalled capillary blood flow in mouse models of ET and PV [43], ischemia, and infarction, including silent microinfarctions in the cerebral microcirculation. Second, the circulating blood cells in MPN patients are constitutively activated with enhanced adherence to each other and the inflamed endothelium [8]. Third, the MPNs are associated with a sustained chronic inflammatory and oxidative stress state, which ultimately give rise to organ dysfunction and organ failure [3–7]. Although platelet activation and elevated levels of inflammatory cytokines, reflecting a chronic inflammatory state are common for both MPNs and AD, neither platelet activation per se nor chronic inflammation per se need to be causally involved in AD pathogenesis, since these conditions are recorded in several other diseases. However, given AD is so common and age-related it is indeed intriguing to consider a causality between platelet activation, chronic inflammation, and AD, since they are all tightly associated with aging (inflammaging/immunoaging). Fourth, a most recent study has unraveled sticking of neutrophils in cerebral capillaries—“stalling” of neutrophils—as one of the main cellular mechanisms, accounting for reduced cerebral blood flow in AD models [44]. As addressed above, similar stalling has been shown in cerebral capillaries of MPN mouse models [43]. Fifth, the link between DS, disordered congenital hematopoiesis, involving the myeloid cell lineage and early development of AD only adds further evidence to the concept that MPNs may be “A Human Neuroinflammation Model” for AD development.

The perspectives of an association between MPNs and AD are several. First, the hypothesis calls for MPN-mutation screening studies among patients with AD and in patient populations which we know have an increased risk of AD as well. Second, considering that treatment with interferon-alpha2 (IFN) normalizes elevated blood cell counts within weeks in the large majority of patients with early stage MPNs (ET, PV, and hyperproliferative myelofibrosis) [63] and interferon-beta most recently has shown potent anti-inflammatory activity in concert with improvement in cognitive function in AD patients [64] and in a rat model [65], studies of the safety and efficacy of interferon-alpha or beta in a larger series of AD patients, preferentially those with early stage disease, might be timely and relevant taking into account that no agents so far have been able to revert disease activity in this devastating disease. Of note, IFN treatment may also impact key factors of utmost pathogenetic importance for AD development, including downregulation of upregulated oxidative stress genes and upregulation of downregulated antioxidative defence genes [66]. Furthermore, IFN also significantly downregulates several upregulated thromboinflammatory genes, not least PADI4, which enhances thrombosis development [67]. Combination therapy with IFN and ruxolitinib—a highly potent anti-inflammatory agent that induces a rapid decline in elevated inflammatory cytokines in concert with alleviation of constitutional symptoms—might also be an attractive approach, since this drug combination has displayed synergistic effects in MPN patients (Fig. 2) [68]. In patients with early stage AD and no MPN disease, treatment with the cytoreductive agent hydroxyurea might be highly interesting, since it has been used successfully for decades in the treatment of patients with sickle cell anemia to reduce the serious complications to this disease, including infarctions in several organs owing to protrombogenic changes in blood cells—very similar to those encountered in patients with MPNs, in whom hydroxyurea worldwide is the most used agent to prohibit thrombotic events. Adding to this, hydroxyurea is not expensive and without safety concerns in patients not suffering a malignant stem cell disease (concern in regard to its leukemogenic potential in patients with MPNs and therefore not used in younger patients).

## Conclusion

In conclusion, based upon a review of shared mechanisms for development of the chronic MPN blood cancers and Alzheimer’s disease and for reduction in cerebral blood flow, including chronic inflammation, involvement of activated myeloid blood cells, and capillary stalling with plugging of blood cells in the cerebral microcirculation, we believe that the hypothesis on MPNs as a “Human Neuroinflammation Model” for AD is worthy to pursue in the future by experimental studies in *JAK2V617F* knock-in mice together with mutation screening of AD patients and patients at high risk of housing MPN-mutations and not least epidemiological registry studies. Indeed, the most recent findings of a frequency of the *JAK2V617F* mutation in stroke patients of 8.3% [69] and in the background population of 3%, implying 10,000 undiagnosed MPNs in Denmark [11] only underscore and put in perspective the urgent need of such mutation screening studies in patients with AD as well. If our hypothesis is supported by results from the above studies, a novel therapeutic approach deserves to be tested in clinical trials addressing the safety and efficacy of monotherapy with IFN or in combination with ruxolitinib in AD. Such a combination therapy is even more timely in the perspective of a revival of the role of blood cells—leukocytes and platelets—in AD pathogenesis and the role of inflammasomes in neuroimmune and neurodegenerative diseases, targeting leukocytes and platelets potently by IFN and the inflammasome by potent anti-inflammatory JAK1-2 inhibition—ruxolitinib—or monoclonal antibodies targeting IL-1beta or IL-6 [70]. By targeting key drivers and the suggested roots of AD development - circulating blood cells - aiming to normalize leukocyte and platelet counts either reactive to chronic inflammatory diseases or clonal (MPNs)—by monotherapies (interferon or hydroxyurea) or in combination with, e.g., ruxolitinib (Fig. 2), such efforts seem more rational than trials, which target single components in AD development, e.g., antibodies targeting beta amyloid or chemokine receptors on leukocytes and platelets [62].

## Data Availability

Not applicable

## Abbreviations

Aβ: Amyloid β-peptide
AD: Alzheimer’s disease
AMD: Age-related macular degeneration
AMKL: Acute megakaryoblastic leukemia
APP: Amyloid precursor protein
BBB: Blood-brain barrier
CAA: Cerebral amyloid angiopathy
CALR: Calreticulin
CNS: Central nervous system
DM: Diabetes mellitus
DS: Down’s syndrome
ET: Essential thrombocythemia
IBD: Inflammatory bowel disease
IFN: Interferon-alpha2, interferon-beta
JAK2: Janus kinase 2
JAK-STAT: Janus kinase-signal transducer and transcription
MF: myelofibrosis
MPL: Myeloproliferative leukemia virus
MPNs: Myeloproliferative neoplasms
NET: Neutrophil extracellular trap
NETosis: NET formation
PV: Polycythemia vera
RBC: Red blood cell
RBSs: Red blood cells
ROS: Reactive oxygen species
TMD: Transient myeloproliferative disorder
VEGF: Vascular endothelial growth factor
